# Salmagundi

**Published:** 1884-11

**Authors:** 


### SALMAGUNDI.

EDITED BY E. G. BETTY, D.D.S.

### POST MORTEM DIFFUSION OF ARSENIC.

Drs. Vaughan and Dawson, of the University of Michigan, have recently conducted some important experiments with the view of ascertaining if arsenious acid injected into the mouth or rectum after death would diffuse through the body. These observers not only found that such was the case, but that the diffusion was very extensive. The results of their investigations have, says the *Lancet*, a very important bearing on the question of arsenical poisoning. In the first place, it can no longer be contended that, because arsenic is found in quantity in the fluids and tissues of the body, therefore death was due to its administration; and in the second, a certain amount of immunity is given to the would-be murderer, inasmuch as there is the possibility of covering a homicidal act by using arsenic with the ostensible purpose of preserving or embalming the body. We say possibility, for such a procedure would almost to a certainty be defeated in its aim. At any rate, there would be no chance of success if the post mortem examination were conducted within a short time of death, when there would be the usual signs of inflammatory action in the alimentary canal; and again, in the face of other circumstantial evidence, the fact of the accused having resorted to such a particular mode of preserving the body would rather tend to confirm suspicion than remove it.

That arsenic contained in soil may be dissolved in water and conveyed into the body has long been known. The researches of Drs. Vaughan and Dawson show what appears *a priori* as probable. During decomposition the relative humidity of different parts of the body, and of these with surrounding media, is constantly changing. Interstitial currents are passing through the tissues by osmotic action, and this liquid diffusion is naturally increased by the presence of crystalloid substances in solution; nor does it cease until the dialysis ends in an equilibrium of attraction which one fluid has for another, or presumably until post mortem disintegration is complete.—*Exchange.*

We offer the above with the hope that it may throw some light upon that frog story with which nearly every one is familiar and if it suggests any new ideas to any one we shall be glad to ventilate them.

---

HYDROGEN may be liquified at a temperature of −371° Fahr.; pretty cold, that.

---

A DOCTOR writes asking the renewal of a bill, and says: "We are in a horrible crisis; there is not a sick man in the district."

---

FROM a careful survey of various doctors' bills the *Merchant Traveler* is strengthened in its faith in the divine injunction: "Physician heel thyself."—*Daily Paper.*

---

PROF. HIRSCH, Berlin, has traced the history of small-pox as far back as the 10th century, and says that the starting point of the disease was in India and Central Africa.

---

A PHYSICIAN writes to the *Boston Globe* that the fumes of tar and spirits of turpentine, in equal parts, burned in an iron pan or dish once in two hours, will cure diphtheria.—*Ibid.*

DAME rumor hath it that our beloved friend and classmate, Dr. N. S. Hoff, of Cincinnati, has gone East to be married. He has the best wishes of his many friends in and out of the profession.

---

WE shall be glad to hear from any or all of our readers regarding their experience with the Per Oxide of Hydrogen. It is a valuable remedy, and it would be gratifying to have some statistical information upon its use and effects.

---

WE have recently seen it stated by good authority that, bran bread is not digested in the human system, the bran passing out without leaving behind it any of the lime salts for which it is recommended. Dr. Berry's "sawdust pudding" has had its day.

---

### DEFINITION OF A DENTIST.

"A dentist, love, makes teeth of bone,
For those whom fate has left without;
And finds provision for his *own*,
By pulling other people's out."—*Scrapbook.*

---

A SO-CALLED "report" of the 24th Annual Meeting of the American Dental Association in "*The Herald of Dentistry*," for October, is an insult to that body and a disgrace to professional journalism. The author must have thought so himself for he forgot to father it with his name.

---

DR. P. ALBRECHT, of Brussels, publishes investigations leading him to conclude that there are primitively four intermaxillary bones in the front of the lower jaw, and that hare-lip, which is never central, takes place by the separation of the inner and outer intermaxillaries of one or the other side. As proof of this, he figures cases of double hare-lips in which the two inner bones stand out as a separate bone, solidified together, while each outer intermaxillary, barring its incisor, is united to the maxillary.—*Leslie's Monthly.*

---

SUTURE OF NERVES.—The report that has just appeared to the effect that M. Tillaux has communicated to the Academy of Sciences the successful suture of nerve in two cases, and that in one case function has been restored in a nerve divided for a period of fifteen years, is, if confirmed, one of the most important facts we have had presented to us in our day. The physiologist, not less than the surgeon, will be led to important work by this event, and fresh fields of inquiry relative to nerve conduction may open new and unexpected advances in the theory as well as the practice of the medical art.—*Scientific American.*

